# An exploratory study of Muslim adolescents' views on sexuality: Implications for sex education and prevention

**DOI:** 10.1186/1471-2458-10-533

**Published:** 2010-09-05

**Authors:** Chris Smerecnik, Herman Schaalma, Kok Gerjo, Suzanne Meijer, Jos Poelman

**Affiliations:** 1 Department of Health Promotion, Faculty of Health, Medicine and Life Sciences, Maastricht University, The Netherlands; 2 School for Public Health and Primary Care (CAPHRI), Maastricht University, The Netherlands; 3 Department of Psychology, Faculty of Psychology and Neuroscience, Maastricht University, The Netherlands; 4 STI-AIDS Netherlands, The Netherlands

## Abstract

**Background:**

This paper describes the results of an exploratory qualitative study on Muslim adolescents' views on sexuality in the Netherlands.

**Methods:**

Data were gathered from an Internet forum on which 44 Muslim and 33 non-Muslim adolescents discussed sexuality as it relates to Islam. These discussions were subsequently analyzed for content using Nvivo 2.0.

**Results:**

Our analysis revealed several issues that are relevant for the design of future sex education programs targeting Muslim youth. Apart from some expected outcomes regarding, for example, taboos on sexuality, sex outside marriage, abortion, homosexuality and conservative gender roles, our analyses showed that in cases of disputes 1) discussions were polarized, 2) opponents used the same Qur'anic passages to support their views, and 3) the authority of an Imam was questioned when his interpretation of Qur'anic passages was not in line with the views of participants.

**Conclusions:**

Our findings show that current approaches to sex education among Muslim youth are likely to be unsuccessful given the rigidity of sexual norms in Muslim society. In addition, we also identified new barriers to sex education among Muslim youth (e.g. lack of respect for an Imam who opposes a youth's views on sexuality).

## Background

The increasing cultural and religious diversity of European societies [1] has established the urgent need for health promotion programs capable of effectively changing adolescents' sexual behavior in a multicultural and multi-religious setting [2,3]. The need for such health programs is further warranted due to the larger prevalence of sexually transmitted infections (STIs) among non-Western immigrants compared with nationals [4,5]. In no other religion or culture is sexuality so closely integrated with religious rules as in Islam [6]. Since health promotion programs are most effective when based on both theoretical notions and empirical evidence [3,7], it is imperative to identify these rules and their influence on the views that Muslim adolescents have on sexuality. Although researchers have examined how Islam influences Muslim adolescents' views on sexuality [e.g., [6,8-10], large gaps still exist in our knowledge about the relationship between Islamic thought and sexuality. The present paper describes an exploratory study on the relationship between Islam and sexuality in the Netherlands.

### Islam and sexuality

Islam has seen rapid expansion throughout the European mainland in recent decades. At present, 5% of the Dutch population aged 18 years and above are Muslim. The majority of these young individuals are of Moroccan and Turkish descent [11]. Religious thought is of enormous influence on believers' views and opinions concerning sexuality [12,13]. Sexuality in Islam is not restricted to procreation as in most other monotheistic religions. Instead, sexuality is considered to be an expression of spirituality. However, Islam does differentiate between 'legitimate' and 'illegitimate' sexuality (referred to as *nikah *and *zinah *respectively), based on marital status [14]. Sexuality within marriage is permitted and socially accepted; sexuality outside marriage is prohibited and socially unacceptable [6,14]. As a consequence, sexuality is not only subject to religious rules, but also has consequences for people's social, economic and public status [14].

Research in the Netherlands has shown that Muslim girls and women up to the age of 25 have little experience with sexuality compared with their Christian and non-religious counterparts. Muslim males, on the other hand, are relatively experienced with sexuality compared with Christian and non-religious peers [15]. Furthermore, Muslim girls are less knowledgeable about sexuality than their non-Muslim peers [16].

Other research has shown that immigration can cause Muslims to challenge their existing views on sexuality [8,17]. Huang and Akhtar [18] argue that immigration can lead to changes in the relative importance of sexuality in daily life, as well as views on homosexuality, marriage, and cross-cultural or cross-religious relationships. As a result of contact with other cultures, the views that immigrant Muslims have on sexuality and sexual decision-making tend to become more individualistic [17,19]. However, although immigration tends to change Muslims' views on sexuality [8,17,18], this does not mean that sex education and prevention programs targeting these Muslims do not require special attention. For instance, conflicts between existing and new views of sexuality may prove 'positive' as well as 'negative' for safe sex practices [18].

Although the relationship between Islam and attitudes toward sexuality and sexual behavior has recently received increasing attention, there are still large caveats in our understanding of this relationship as well as its implications for sex education and prevention programs. This may at least in part be explained by the fact that Muslim adolescents are relatively reluctant to participate in studies about sexuality. Indeed, Muslim participation has been rather limited in conventional survey studies [15]. As a consequence, the focus has shifted to a more indirect way of mobilizing Muslim adolescents for research by using the Internet. More specifically, Internet forums, which can be considered a type of focus group interview, were used to this end in the present study.

### Internet and research

As a fairly new and valuable method available to health practitioners and educators, the Internet has potentially unlimited dissemination, accessibility and possibilities [20,21]. Tentative steps have recently been taken towards using the Internet in interventions and quantitative research [22-25]. The value of using the realm of the Internet for qualitative research has also been recognized [26-28]. Specifically, Internet research can be used to investigate otherwise hard-to-reach populations [26], such as Muslim adolescents [15]. Using data gathered from an Internet forum, the present study also adds to our insights about the applicability of Internet forum discussions to study attitudes and opinions concerning controversial issues like Islam and sexuality.

### Overview

The present study aims to explore the relationship between Islamic thought and views on sexuality among Muslim adolescents in the Netherlands. We focus on the following research questions. First, what are Muslim adolescents' current views on sexuality? By using a sample that differs from those used in previous research, we were interested in whether we could replicate previous findings or identify any new views. Second, how and to what extent do these views differ from the views of non-Muslim adolescents? By comparing Muslim adolescents' views with those of non-Muslims we expected to identify 'problematic' views (in the sense that they may impede sex education efforts) with more ease. Third, we hoped to learn whether existing approaches to sex education among Muslim adolescents are likely to be successful, and whether new approaches could be identified. In this exploratory study, we used data on sexuality collected from an Internet discussion forum. We did not seek ethical approval for this study because the Internet forums were publicly accessible.

## Methods

### Participants

The present exploratory study analyzed the contributions of 77 visitors (44 Muslims; 33 non-Muslims) to the discussions on the publicly accessible Internet forum Islam & Sexuality. In total, 269 participants visited the forum and participated in the discussions that were used in this study. However, since we were not able to determine participants' religious backgrounds beforehand, Muslim and non-Muslim participants were differentiated on the basis of explicit indications in the discussions. In other words, we only categorized participants as being either Muslim or non-Muslim if they explicitly mentioned on the forum that they were Muslim (e.g. 'I'm Muslim') or non-Muslim (e.g. 'I'm Christian'). We categorized 44 participants as Muslim and 33 as non-Muslim. It is important to note that the 33 'non-Muslim' participants concern non-Muslim immigrants as well as Dutch adolescents who are not Muslim. In the same manner, the 44 'Muslim' participants include both Muslim immigrants and Dutch adolescents of Islamic faith. For the sake of brevity, we use the terms 'non-Muslim' and 'Muslim' for these groups.

### Procedure

As stated, the data analyzed in the present study stem from discussions posted on the Internet forum Islam & Sexuality, which can be found at http://www.maroc.nl. The website was initiated by STI-AIDS Netherlands (Expertise Center for HIV/AIDS and other STIs) with the aim of making sexuality a subject of discussion, and informing visitors about various aspects of sexuality and relationships. One of the website's secondary goals was to support adolescents in making informed choices concerning relationships and sexuality.

The website was initially designed to present Muslim adolescents with a means to learn about healthy sexual relationships and STI prevention and to discuss sexuality as it relates to Islam. Muslim adolescents between the ages of 14 and 24 were the target audience. Besides the forum on Islam & Sexuality, the website offered general information about sexuality, STIs and prevention. Furthermore, visitors were offered the opportunity to ask questions to an Imam or one of the two employees who were involved from the Municipal Public Health Service (GGD). The Imam and the two GGD employees were trained and thus qualified to provide information concerning sexuality and Islam. The Imam and the two GGD employees were not involved in the forum discussion, but instead responded to questions posed on another section of the website.

Visitors to the Islam & Sexuality forum were able to start a discussion by asking a question about sexuality or by posting a statement to which other visitors could respond. We collected all the discussions posted on the forum from June 2004 until September 2005. Before analyzing the data, all personal qualifiers were removed to ensure participant anonymity.

The data were analyzed for content using Nvivo 2.0 [29]. Attention was specifically directed toward differences between Muslim and non-Muslim participants and their implications for sex education and prevention. To analyze the data collected from the internet forum, the first author first coded all instances in the data concerning sexuality, and determined whether these instances were made by Muslim or non-Muslim participants. In the second phase, the topic of the reference was determined (i.e. sex outside marriage, inter-religious relationships, masturbation, homosexuality, and abortion; see Table 1). Subsequently, the content coding was refined to include more specific topics (e.g. we sub-coded the topic masturbation under references concerning satisfaction as a result of masturbation or prevention of *zinah*). In the fourth phase, we coded whether these instances were used as an argument for or against the topic in question (e.g. instances concerning satisfaction as a result of masturbation can either be used as an argument for masturbation in that it gives satisfaction and prevents *zinah *or as an argument against masturbation in that it does not give long-term satisfaction or that it may otherwise negatively influence a person's faith). These analyses were conducted for each topic separately as well as for all topics combined. As a result, it was possible to identify common themes within the views that Muslim adolescents had on sexuality.

**Table 1:** Number of discussions, participants, and messages per topic.

	Number of discussions	Number of participants	Number of messages
Sex outside marriage	26	159	945
Inter-religious relationships	21	110	435
Masturbation	6	35	123
Homosexuality	5	22	118
Abortion	4	11	63

Note. These frequencies represent all posts on the forum and were subsequently analyzed for his study.

## Results

First, we will report the results for each of the discussion topics separately. Subsequently, we will discuss some general observations.

### Sex outside marriage

The discussions about sex outside marriage revealed several differences between Muslims and non-Muslim regarding extramarital sex. Sex before marriage was frowned upon by both male and female Muslims, but generally approved by non-Muslims. Muslim participants argued that sex before marriage is *haram *(a sin) in Islam. As a consequence, they associated sex before marriage with notions of the virginity standard. According to the participants, this standard applies to both male and female Muslims.

The commandment about sex before marriage is told in the Qu'ran from a male's perspective but also applies to women. (Female, the Netherlands, Muslim)

In-depth analysis of the discussions, however, revealed the existence of a 'double morality' regarding sex before marriage - male Muslims generally do not seem to comply with this rule even though they themselves did consider sex before marriage to be *haram*. This, however, did not prevent them from asserting that they should be allowed to have more freedom than female Muslims. Interestingly, male Muslims were not called to account for this transgression; female Muslims seemed to quietly accept this double morality. Moreover, they claimed to guard their own virginity for the sake of these male Muslims who themselves did not bother to remain virgins.

Concerning men it's unfair that I remain a virgin and he doesn't... But I am reconciled to the fact that men are different creatures than women. Even if a guy has fooled around a lot, he will still want a virgin. (Female, the Netherlands, Muslim)

Non-Muslims agreed that sex before marriage is a normal part of every relationship. By having intercourse, partners get to know each other. Not having sex before marriage might lead to sexual frustration within marriage and can lead to divorce.

Good sex is the glue in a relationship, people... You can argue that a relationship is based on love and respect but don't forget that oh so important feature: sex. As a result you may have sexual frustrations in your marriage. So I am a supporter of defloration before marriage... (Female, non-Muslim)

Adultery, on the other hand, was condemned by both Muslims and non-Muslims. It was generally agreed that adultery is not acceptable. Muslims, however, were more extreme in their convictions about adultery than non-Muslims.

Committing zinah [adultery] is a dreadful sin and is not easily forgiven. A lot of people think very lightly about this: I did it and I'll just ask Allah to forgive me!! You shouldn't have such thoughts, the penalty is very clear, for men as well as women!!!!! (Female, Muslim)

### Inter-religious relationships

In initiating and maintaining an inter-religious relationship, Muslim participants considered their parents' opinions of utmost importance. According to Muslim participants, an Islamic marriage is not legal if the parents do not give their blessing to the marriage. In the worst-case scenario, Muslims participants were forced to either give up either their relationships or their families. They generally seemed to accept the power and control of their parents over their marriage - they knew that their parents' attitudes were based on good motives.

I'm Muslim and have a relationship with a Christian boy. I have dated him for 4 years and I love him very much. There is 1 problem though: my parents don't accept him. I don't know which way to turn, I don't want to lose him, but I don't want to lose my parents either. (Female, Muslim)

Hey everybody, I have a question: what would you do if you had to choose between your parents and your boyfriend? I have been in a relationship with him [a non-Muslim] for almost 2 years and he proposed to me but my parents refused him. I don't know why... I'm at an utter loss, I love him and my parents. I don't know what to do... (Female, the Netherlands, Muslim)

Although most Muslim participants indicated that they only wanted to marry another Muslim, the majority of the Muslim participants considered the ethnic background of their future partner to be unimportant, as long as he or she was of the Islamic faith. A minority of Muslim participants did however seem to value cultural heritage.

I mean, honestly, if you have a Dutch husband, he would never understand your culture and you would never understand his. Let me warn you: this will cause arguments and cracks in your relationship. This is why your parents don't want you to date such a man. They do not want your heart broken, because you are their daughter and they love you and they know what's best for you. (Female, Muslim)

Non-Muslims, on the other hand, argued that a partner's religion was not overly important. Instead, they believe that love and happiness are the pillars of a good relationship. To them it was inconceivable that Muslim parents would not approve a relationship in which their child is happy. Furthermore, non-Muslims could not understand the weight that Muslims gave to their parents' opinions when faced by the choice of whether or not to initiate or maintain an inter-religious relationship.

I love my boyfriend so much!! He's a Muslim and his parents will never accept our relationship... I wish his parents would give me a chance to prove myself, or something... But that will probably never happen. Why is religion more important than the happiness of your own children???? (Female, the Netherlands, non-Muslim)

### Masturbation

Muslim participants reluctantly discussed masturbation. In general, they agreed that all sexual acts outside marriage, including masturbation, are *haram*. Muslim participants seemed to extend the restrictions on sex before marriage to masturbation. They argued that masturbation is 'sex with yourself' and that it stems from lust. Furthermore, they argued that masturbation leads to more sin, for instance *zinah *(adultery).

Masturbation may lead to more haram. For instance, it may lead to zinah. (Female, Muslim)

Non-Muslims, on the other hand, clearly distinguished between masturbation and sexual intercourse. They considered masturbation to be perfectly natural, even within relationships. Furthermore, non-Muslim participants argued that masturbation functions as a release of excess sexual tension and, as a consequence, may *prevent *adultery.

Would you prefer that this person [who is not allowed to masturbate] commit adultery (Female, the Netherlands, non-Muslim)

### Homosexuality

All Muslim participants considered homosexuality to be *haram*. Furthermore, since homosexuality is considered *haram*, Muslim participants argued that Allah would not allow someone to be born as homosexual. Following this reasoning, they considered homosexual practices to be a person's own perverse choice; in essence, they blamed the homosexual for *choosing *to live in sin.

You're saying that Allah would allow for someone to be born a homosexual ... and then tell this person not to perform these [homosexual] deeds. Allah does not burden someone above his or her power. (Male, heterosexual, Muslim)

Non-Muslims considered homosexuality to be genetically determined. In their view, homosexual preferences cannot be changed. Furthermore, homosexuals were not considered different from heterosexuals except in sexual orientation.

### Abortion

Many of the discussions about abortion concerned unintended pregnancies. According to the Muslim participants, the mothers-to-be are to blame for their unintended pregnancies; they should have anticipated the consequences before acting upon their impulses or desires. In contrast, non-Muslims argued that this may not always be possible and that not all consequences can be predicted.

What if, what if??? You should have thought of that [the risk of unwanted pregnancy after sexual intercourse]! Say a woman gets pregnant... for the sake of family and honor [she] has to deal with the consequences... (Female, the Netherlands, Muslim)

This quote also illustrates that, according to this Muslim participant, the implications of unintended pregnancies concern the entire family. Non-Muslims participants see this family involvement as complicating an acceptable outcome of the unintended pregnancy.

### General observations

One common theme that could be identified was the differing interpretations of Qur'anic passages. Muslim participants sometimes reported two or more different interpretations of the same passage. However, even when participants agreed on the 'correct' interpretation of the passage, the same passage was used as an argument for two opposing views. For instance, the passage "And who guard their private parts, except before their mates or those whom their right hands possess, for they surely are not to blame, but whoever seeks to go beyond that, these are they that exceed the limits" (23:5-6-7) was used to argue that masturbation is prohibited by the Qur'an as well as to argue that it is not explicitly mentioned and thus not prohibited. Although *the *correct interpretation may not exist - these interpretations represent two of the many explanations that there may be for an ambiguous passage - it was obvious from the discussions that these different interpretations are the cause of much uncertainty and confusion among Muslims. The Qu'ran passage quoted above, for instance, was explained to mean that

you (both men and women as the commandments about sex and marriage apply to both men and women) should not guard your chastity towards your spouse and slaves, but if you wish more than that (meaning you seek your satisfaction somewhere else... with another woman or yourself!) you are committing a sin. (Female, the Netherlands, Muslim)

but also that

those who transgress against their spouses or those whom their right hands possess are committing zinah. The fact remains that masturbation is not mentioned in this passage. (Female, Ajman)

A related issue is the observation that when the Imam on the forum attempted to clarify interpretations of the Qur'an, several Muslim participants seemed to question his authority and expertise. For instance, when the Imam stated that Islam was not as conservative and restricting regarding sexuality as most Muslim participants seemed to assume, many characterized the Imam's explanation as a false interpretation of the Qur'an, whereas they considered their own 'interpretation' to reflect the Qu'ran's true meaning. The word of an Imam is, apparently, not by definition law. One Muslim even stopped referring to him as 'Imam' and instead addressed him as 'Mr.'.

[Imam], I don't know where you have been studying, but this is incorrect. Moreover, you don't present evidence and detail (Female, Morocco, Muslim)

[Imam], my judgment about you: you have gone astray, ignorant (Male, France, Muslim)

Finally, we observed that Muslim participants were generally quite reluctant to discuss sexuality on the Internet forum. They seemed to place great value on modesty and privacy, and argued that questions about sexuality are socially unacceptable. Furthermore, some Muslim participants stated that questions about sexuality are useless because every 'real' Muslim already knows the answers - the Qur'an offers guidelines for every Muslim, and consequently it is redundant to look for answers elsewhere.

I think this question is a very weird one; we all know it is prohibited. So people, you should not be asking these kinds of questions (Female, the Netherlands, Muslim)

## Discussion

In this exploratory study, we used data gathered from an Internet forum to explore the relationship between Islam and Muslim adolescents' views on sexuality. Our analyses of the Internet discussions about Islam and sexuality enabled us to gather information that may prove useful for the development of future sex education and prevention programs. The analyses revealed a number of differences between Muslim and non-Muslim adolescents in terms of their views on sexuality. In general, our results confirm previous findings on Muslims' views on sexuality. Considering that we used a largely unexplored sample (i.e. participants on an internet forum), our results could be seen as having increased the validity of previous findings.

However, one finding clearly stands out and - to our knowledge - has not been observed in previous research: the notion that Muslim adolescents cannot necessarily persuaded by an Imam. Some of our participants actually denounced the Imam on the forum as unknowledgeable when he contradicted the participants' worldviews. This finding has consequences for sex education among Muslim adolescents. Since sexuality is closely integrated with religious rules [6], involving the Imam in sex education programs would seem an effective strategy to counter Muslim adolescents' rigid views on sexuality. However, this may not actually be the case for subpopulations with extreme views, as these Muslim adolescents are more likely to denounce the religious authority of a liberal Imam than change their views on sexuality. This does not mean that an Imam's active involvement in sex education is not suitable for a large minority or even a majority of Muslim adolescents with more open-minded views on sexuality. We do suggest caution when involving an Imam in sex education programs and urge researchers to examine the Muslim adolescents' perceptions of, and reactions to, a liberal Imam beforehand.

As observed in previous research [30], the forum discussions underline the relevance of Islam for the daily life of many participants. These forums further revealed that the respondents identified as Muslim tend to respect their cultural heritage, and that culture and religion may be intermingled for immigrant Muslims. Indeed, in Morocco, Muslim youth adhere to global trends but are still rooted in local thought [30]. One challenge of future research will be to explore whether distinguishing between Islamic rules and cultural values is a useful strategy to promote sexual health among target populations with an Islamic background.

Our study further supported the general finding in previous research that sex before and outside marriage - and even masturbation - is condemned among Muslims. However, this condemnation does not guarantee adherence to these rules, and ignorance about sexuality and sexual risks may adversely influence safe sex practices during Muslim adolescents' first contact with sex. Consequently, there is a real need to inform and prepare Muslim adolescents for their first experience with sexual intercourse. Future research should explore how Islamic views on sexuality can be integrated in comprehensive sex education, and whether promoting liberal interpretations of the Qur'an permitting contraceptive use and protective measures is a realistic option to educate Muslims adolescents about sexuality and safe sex practices. As observed in our study, a strong dilemma for such an approach concerns the tendency of some to deny and condemn opposing viewpoints.

Many participants also reported experiencing some difficulties with initiating and maintaining inter-religious relationships and dealing with parental disapproval of such relationships. Since marriage in Islam is not considered to be legitimate without parental approval, many Muslim adolescents feel that they are forced to choose between their partner and their parents. The importance of family influence was also observed in the discussions about abortion. Since health decisions - including issues like unintended pregnancy - concern the entire family and are rarely made without consulting the family [10], it is hard to see how to develop comprehensive sexuality education for Muslim youth without involving parents, and perhaps the entire family. Given the general difficulties with involving parents in sexuality education [31], the lack of parent-child communication about sexuality among Muslims [32], and the negative views held by Muslim parents on school-based sexuality education [33], it has been suggested that parents should be actively involved in the process of development, implementation and monitoring of school-based sexuality education [34]. Indeed, although parent-child communication was primarily restricted to the risks posed to the social order by premarital sexual relationships, Muslim girls preferred to receive sexual education from their mothers, and felt that school-based sexual education programs marginalized the influence of Islam on choices concerning sexuality [32]. As a consequence of this perceived marginalization of Islam, some Muslim girls withdrew from the school-based sex education programs [32]. Teaching Muslim parents about sexuality and how to educate their children may improve sexual education among Muslim adolescents. Since sexuality education is segregated in Islam [35-37], such a strategy would have to involve both fathers and mothers.

However, given our findings on the rigidity of some Muslim adolescents' views on sexuality, we wonder whether such approaches would be successful. Firstly, our results seem to suggest that most Muslim adolescents are generally unwilling to discuss sexuality. We have no reason to believe that adolescents would be more inclined to discuss sexuality with their parent than with their peers. Secondly, we saw that Muslim adolescents denounced an Imam when they did not agree with his (more liberal) views on sexuality. Would they be more willing to be persuaded by their parents?

The forum discussions in our study further revealed views on sexuality that were strongly gender-based. Muslim women and girls, for instance, generally regarded the guidelines in the Qur'an regarding sex and marriage to be relevant for males and females, whereas Muslim men and boys tended to regard these rules to be relevant only for women. Consequently, many men seem to have sexual intercourse with women from different ethnic groups before they start looking for a potential spouse within their own ethnic group [9]. In addition, male Muslim participants seemed to be more provocative than female Muslims in their reactions to questions and issues that were raised by other participants. When confronted by a serious question, male Muslim participants tended to answer in a sarcastic, provocative way more often than female Muslims. It will be a real, but necessary, challenge for sex education to incorporate this 'double morality' that seems so deeply rooted in cultural and religious norms and values [9] into a comprehensive sex education and prevention program targeted at Muslim adolescents.

Perhaps one of the most interesting findings of this study concerns the confusion about the interpretation of Qur'anic passages that implicitly address sexuality, and the polarized nature of subsequent discussions. Although the problem of differing interpretations about ambiguous passages is by no means unique to Islam and may be inherent in any religion, it might also reflect the changing domain of sexuality among Muslims as a result of the discrepancies between Islamic doctrine and its application in real life [30]. Indeed, the present results show that, in the case of a dispute, participants who disagreed with one another used the same passages to back their opposing viewpoints. In addition, Muslim participants occasionally questioned the authority and expertise of the Imam when his interpretation contradicted their own opinions about sexuality-related issues. When opponents quoted the Imam on a particular subject, some Muslim adolescents vehemently denied the expertise of the Imam.

Both the nature of the discussions and the distrust in the Imam are important for the development of sex education targeting Muslim youth. Efforts to educate Muslim adolescents about sexuality and safe sex practices have already been undertaken. However, our results suggest that these efforts may not be successful given the strong norms in Muslim society. One such effort, sexuality education in classrooms, depends upon the establishment of a 'safe' classroom atmosphere - young people should feel free to discuss intimate issues because discussing sexual behavior may break taboos concerning the public discussion of sexuality. Given the nature of the discussions that we observed on the Internet forum, the creation of such a safe atmosphere will need special attention and may require intensive ground rules and feelings-and-values exercises, in which mutual respect, embarrassment and its effects, and the limits of acceptable disclosure are discussed. A second possible strategy, the active involvement of religious leaders in educating Muslim youth about sexuality, may not be a 'magic bullet' in introducing comprehensive sex education to Muslim youth, especially when these adolescents are part of a multi-cultural and multi-ethnic community. Based on our findings, we know that current approaches to sex education among Muslim adolescents are likely to fail. In addition, common-sense approaches such as involving an Imam may not be entirely successful either, given that Muslims may question an Imam's authority when presented with an opposing worldview.

An additional problem for sex education targeting Muslim youth seems to be the general reluctance to discuss sexuality. This reluctance to discuss sexuality seems to be related to the striking absence of discussions about safe sex practices, such as condom use. As mentioned before, the Internet forum Sexuality & Islam was specifically developed to provide a platform for a safe and anonymous discussion about safe sex and condom use. However, discussions about safe sex were not observed on the forum. Muslim participants' reluctance to ask questions about, and discuss sexuality may at least partly explain the lack of discussion of the controversial topics of safe sex practices and condom and contraceptive use. Participants in our discussions frequently argued that questions and discussions about sexuality are useless because the Qur'an offers the guidelines that every Muslim should follow - in their view there was no need to ask questions or to look for answers other than those provided by the Qur'an [14]. Since effective sex education programs are based upon active learning and participation and include group discussions and small group work [38], it is important to find strategies to solve this dilemma.

Our findings confirm the existence of previously observed barriers to successful sex education among Muslim adolescents, and also highlight some new barriers. Future research is needed to identify the possibility of involving Imams in the development and delivery of sex education programs, and what role Imams should play. In addition, research is needed into effective strategies to educate Muslim adolescents about sexuality and safe sex practices.

We were also interested in the extent to which Internet forums can be used as a research tool to gather information about issues that are considered controversial. We assumed that using data posted on an Internet forum would reduce, if not eliminate, participants' confirmation bias, since individuals might feel more secure due to perceived anonymity and *casu quo *might be more willing to honestly express their views and opinions. In addition, Internet samples might be more diverse than samples in usual offline research, such as interviews and surveys [39,40]. Internet samples differ from paper-and-pencil samples, but there is no consensus on which type of sample provides a better representation of the population. Gosling et al. [39] compared Internet and paper-and-pencil samples and concluded that Internet samples are more diverse and are not adversely affected by non-serious or repeat responders, and that Internet findings are consistent with findings from more traditional methods. Indeed, several studies have compared Internet studies with similar studies using more conventional methods and have shown that the results are largely comparable [41]. Many of our findings are compatible with the findings of other studies, while some are not. Where our findings vary, sampling differences may be the cause, but it is not immediately clear which sample provides the best representation of the total population.

On the other hand, the use of Internet forums as a research tool has a number of disadvantages. For instance, Internet research is subject to two selection biases: the non-representative nature of the Internet population itself and the self-selection or 'volunteer' bias [42]. These selection biases raise questions about the validity of generalization [42]. Furthermore, researchers have limited control over the course of the discussion, and it is difficult, if not impossible, to identify and verify participants' personal characteristics. The present results should therefore be cautiously interpreted in light of the restrictions that these limitations pose to the generalizability of the results.

Despite these limitations of Internet research, the present study suggests that Internet forums can be useful tools for studying controversial issues among 'hard-to-reach' populations - in this case sexuality and Islamic youth. The results of this study identified issues that are important for the development and implementation of future sex education programs targeting Muslim youth. As mentioned above, using Internet discussions to identify needs, opinions and controversies, however, also has serious drawbacks and limitations. These primarily concern the identification of participants - participants are genuinely anonymous and can easily misrepresent themselves in any way they like. A disadvantage of the forum format that we used is that researchers cannot actively raise discussion topics. It therefore seems important to apply Internet discussions in combination with traditional qualitative and quantitative studies to enable triangulation of research findings.

Whereas the anonymity of participants is a major limitation of using Internet forums, it is also one of the major strengths since it facilitates discussions on issues that are regarded taboo. Although Muslim adolescents still seem reluctant to address and discuss sexuality even in the relative safety of the Internet, using the Internet would provide researchers and educators with the opportunity to reach Muslim adolescents and provide them with information on safe sex practices. As such, the Internet may also be an important medium to deliver future sex education programs.

## Conclusions

The present study confirmed previous findings on the views held by Muslim adolescents on sexuality, such as the 'double morality' and views on masturbation and sex outside marriage. One important new finding was that some Muslim adolescents actually denounced an Imam as unknowledgeable when the Imam contradicted their worldviews. This finding suggests caution when involving Imams in sex education programs. Further research is needed to examine in more detail Muslim adolescents' perceptions of, and reactions to, the participation of liberal Imams in sex education programs.

## Competing interests

The authors declare that they have no competing interests.

## Authors' contributions

CS analyzed and interpreted the data and was responsible for drafting the manuscript. HS and GK helped interpret the data and supervized the writing of the manuscript. SM and JP assisted in writing the manuscript. All authors read and approved the final manuscript.

## Pre-publication history

The pre-publication history for this paper can be accessed here:

http://www.biomedcentral.com/1471-2458/10/533/prepub
